# Genomic signature of parity in the breast of premenopausal women

**DOI:** 10.1186/s13058-019-1128-x

**Published:** 2019-03-28

**Authors:** Julia Santucci-Pereira, Anne Zeleniuch-Jacquotte, Yelena Afanasyeva, Hua Zhong, Michael Slifker, Suraj Peri, Eric A. Ross, Ricardo López de Cicco, Yubo Zhai, Theresa Nguyen, Fathima Sheriff, Irma H. Russo, Yanrong Su, Alan A. Arslan, Pal Bordas, Per Lenner, Janet Åhman, Anna Stina Landström Eriksson, Robert Johansson, Göran Hallmans, Paolo Toniolo, Jose Russo

**Affiliations:** 1 The Irma H. Russo, MD Breast Cancer Research Laboratory, Fox Chase Cancer Center - Temple University Health System, 333 Cottman Ave, P2051, Philadelphia, PA 19111 USA; 2 Division of Epidemiology, Department of Population Health, New York University School of Medicine, New York, NY 10016 USA; 3 New York University Perlmutter Cancer Center, New York, NY 10016 USA; 4 Department of Biostatistics and Bioinformatics, Fox Chase Cancer Center - Temple University Health System, Philadelphia, PA 19111 USA; 5 Department of Obstetrics and Gynecology, New York University School of Medicine, New York, NY 10016 USA; 6 Sunderby Hospital, Luleå and the Norrbotten Mammography Screening Program, Luleå, Sweden; 7 Departments of Radiation Sciences and Oncology, Umeå University, Umeå, Sweden; 8 Department of Biobank Research, Umeå University, Umeå, Sweden; 9 Department of Public Health and Clinical Medicine, Umeå University, Umeå, Sweden

**Keywords:** Gene expression profiling, Pregnancy, Breast differentiation, Parous and nulliparous breast transcriptome, Immune response, Breast cancer risk, Normal breast transcriptome

## Abstract

**Background:**

Full-term pregnancy (FTP) at an early age confers long-term protection against breast cancer. Previously, we reported that a FTP imprints a specific gene expression profile in the breast of postmenopausal women. Herein, we evaluated gene expression changes induced by parity in the breast of premenopausal women.

**Methods:**

Gene expression profiling of normal breast tissue from 30 nulliparous (NP) and 79 parous (P) premenopausal volunteers was performed using Affymetrix microarrays. In addition to a discovery/validation analysis, we conducted an analysis of gene expression differences in P vs. NP women as a function of time since last FTP. Finally, a laser capture microdissection substudy was performed to compare the gene expression profile in the whole breast biopsy with that in the epithelial and stromal tissues.

**Results:**

Discovery/validation analysis identified 43 differentially expressed genes in P vs. NP breast. Analysis of expression as a function of time since FTP revealed 286 differentially expressed genes (238 up- and 48 downregulated) comparing all P vs. all NP, and/or P women whose last FTP was less than 5 years before biopsy vs. all NP women. The upregulated genes showed three expression patterns: (1) transient: genes upregulated after FTP but whose expression levels returned to NP levels. These genes were mainly related to immune response, specifically activation of T cells. (2) Long-term changing: genes upregulated following FTP, whose expression levels decreased with increasing time since FTP but did not return to NP levels. These were related to immune response and development. (3) Long-term constant: genes that remained upregulated in parous compared to nulliparous breast, independently of time since FTP. These were mainly involved in development/cell differentiation processes, and also chromatin remodeling. Lastly, we found that the gene expression in whole tissue was a weighted average of the expression in epithelial and stromal tissues.

**Conclusions:**

Genes transiently activated by FTP may have a role in protecting the mammary gland against neoplastically transformed cells through activation of T cells. Furthermore, chromatin remodeling and cell differentiation, represented by the genes that are maintained upregulated long after the FTP, may be responsible for the lasting preventive effect against breast cancer.

**Electronic supplementary material:**

The online version of this article (10.1186/s13058-019-1128-x) contains supplementary material, which is available to authorized users.

## Background

The association of parity with breast cancer risk is well documented by both epidemiological and experimental data [1–4]. While the relationship is complex, with a transient increase in risk after each full-term pregnancy (FTP), the long-term effect for women who have their first FTP at an early age is a marked reduction in risk [5]. A better understanding of the molecular mechanisms underlying the effects of parity on the breast may help develop strategies to prevent breast cancer.

We previously reported the results of a study that assessed gene expression differences in the breast of 67 parous (P) and 40 nulliparous (NP) postmenopausal women who were free of any pathology and had volunteered to undergo a tissue biopsy [6–8]. We reported that in the postmenopausal breast, parity-induced gene expression changes were related to differentiation of this organ [6]. More specifically, we found that genes upregulated in the P breast, as compared to the NP breast, represented biological processes involved in differentiation and development, cell junction, RNA metabolic processes, and splicing machinery. The downregulated genes represented biological processes involved in cell proliferation, regulation of IGF-like growth factor receptor signaling, somatic stem cell maintenance, muscle cell differentiation, and apoptosis [6, 7].

We here report on a study with a similar design and conducted in the same general population, but focusing on premenopausal women. The main objective of this study was to assess the parity-associated gene expression differences in the breast of premenopausal women. Because the breast undergoes involution after pregnancy and there is a short-term increase in breast cancer risk following each FTP, we examined the gene expression differences in P vs. NP women as a function of time since last FTP. Additionally, we conducted a substudy, in which laser capture microdissection (LCM) was used to isolate breast epithelial cells from the stroma to evaluate how the gene expression observed in RNA extracted from whole breast tissue relates to gene expression in RNA extracted from breast epithelial cells and from stroma separately.

## Methods

### Study population and eligibility criteria

Study subjects were volunteers recruited among healthy women between the ages of 29 and 47 years and residing in Norrbotten County, Sweden. Exclusion criteria included a history of any cancer, complete bilateral oophorectomy, breast biopsy or breast implants, and hormonal treatment for infertility. Women who had completed a FTP or breastfed in the 12 months prior to enrollment, used oral contraceptives in the 6 months prior to enrollment, or used thyroid or steroid hormones, anti-coagulants, or diabetes medications in the 3 months prior to enrollment were also ineligible. The study was approved by the Regional Ethical Review Board for Northern Sweden at the University of Umeå, Sweden.

Volunteers who signed informed consent were scheduled for a biopsy. Women who had not had a mammogram within the year preceding enrollment received one prior to the biopsy to exclude breast cancer. Parous (P) women were defined as women who had had one or more full-term pregnancies, defined as a pregnancy lasting at least 37 weeks. The nulliparous (NP) group included women who had never been pregnant or who had no history of pregnancies lasting more than 8 weeks.

### Data and breast tissue collection

Eligible volunteers completed a questionnaire that collected data on reproductive history, medical history, height and weight, first-degree family history of breast cancer, history of tobacco use, and current medications. Breast core needle biopsies were performed by two experienced radiologists (P. Bordas and A. Eriksson) at the Mammography Department at Sunderby Hospital, Luleå, Sweden. A 12-Gauge BARD® MONOPTY® core biopsy needle was used, and four to eight biopsies were taken from the upper outer quadrant of one breast. One biopsy specimen was placed in 70% ethanol for histopathological analysis, and the remaining in RNALater® (Ambion) solution. Tissue samples were stripped of all personal identifiers and sent to Fox Chase Cancer Center for analysis. All samples were first reviewed by the study pathologist (J. Russo) to confirm the absence of atypia or cancer. During all the experiments, the researchers at Fox Chase Cancer Center were blinded to the parity status of the samples.

### Gene expression microarrays

Total RNA from the biopsies was isolated using the Allprep RNA/DNA Mini Kit (Qiagen, Alameda, CA, USA). Quantity and quality of total RNA were assessed using NanoDrop v3.3.0 (NanoDrop Technologies, Wilmington, DE, USA) and Agilent 2100 Bioanalyzer (Agilent Technologies, CA, USA), respectively.

GeneChip Expression 3′-Amplification Two-Cycle cDNA Synthesis Kit (Affymetrix, Santa Clara, CA) was used for sample preparation and hybridization to Affymetrix Human Genome Gene Chip U133 Plus 2.0 arrays. For quality control purposes, we included in each batch (9 to 12 samples) one blinded duplicate sample from another batch.

After scanning, all microarrays were subjected to quality control (QC) analysis to ensure that they were in the acceptable ranges for standard Affymetrix quality measures (Scale Factor, Percent Present, and Average Background). In addition, quality was assessed using graphical tools based on Affymetrix probe-level models (PLM) [9]. The Normalized Unscaled Standard Error (NUSE) plot, in particular, was used to disqualify lower quality arrays. Ten arrays (8%—9 P and 1 NP) did not fulfill quality criteria and were not included in the statistical analysis. The concordance correlation coefficients for the QC replicates were greater than 98%.

### Data preprocessing and batch adjustment

The Affymetrix data were analyzed using R language for statistical computing (R version 2.14.1) [10] and Bioconductor [11]. Preprocessing methods and filtering criteria were similar to those used in our previous study [6, 7]. Probesets for which both the proportion of present calls was < 75% and the difference in proportion of present calls between P and NP samples was < 25% were filtered out. Probesets with coefficient of variation (CV) across samples falling in the first quartile were also excluded. After filtering, 14,920 probesets (27%) remained in the analysis. To account for inter-batch variability, the data were adjusted for batch using the ComBat method [12] implemented in the Bioconductor package sva [13].

### Statistical analysis of gene expression differences between parous and nulliparous women

Linear regression models were used to identify probesets differentially expressed in P vs. NP samples. For each probeset, an unadjusted *p* value measuring the significance of parity (yes/no) as an independent predictor of the log-transformed normalized gene expression value was calculated using single regression. We also used multiple regression analysis to identify differentially expressed probesets while controlling for potential confounders. The associations of subject characteristics with parity status and with gene expression were examined to identify potential confounders. Multivariate models were adjusted for age, body mass index (BMI) (which was associated with gene expression), and smoking duration (which was associated with both gene expression and parity status). We also adjusted for phase of cycle/use of a hormonal IUD, which could affect gene expression. False discovery rate (FDR) was used to control for multiple comparisons, using QVALUE in the R package version 1.28.0 [14].

In order to identify the most robust parity-associated differences in gene expression, we first analyzed the data using a discovery/validation resampling approach. A discovery dataset was generated by selecting at random 2/3 of the P women and 2/3 of the NP women from the complete dataset. The remaining women formed a corresponding validation dataset. This step was repeated 12 times, leading to 12 discovery/validation dataset pairs. Probesets with FDR < 20% in any discovery dataset and *p* value < 0.05 in the corresponding validation dataset were considered validated for this dataset pair. We report the probesets (and corresponding genes) that were validated in at least 2 of the 12 dataset pairs.

The breast undergoes involution after pregnancy, which is likely to be associated with transient changes in gene expression. Further, although the long-term effect of early parity (before 35 years of age) is a reduction in breast cancer risk for pregnancy [5], it is well documented that there is a short-term increase in risk after each FTP. This suggests that the gene expression pattern in the breast may be different in the first few years after pregnancy than in later years. Therefore, we examined the parity-associated gene expression differences according to time between last FTP and biopsy (time since last pregnancy, TSLP). To optimize our chances of detecting TSLP-related differences in gene expression, we included in these analyses probesets that were differentially expressed (FDR < 10% and at least 1.2-fold change) in the subgroup of P women whose last FTP was ≤ 5 years before biopsy as compared to NP women, in addition to the probesets identified in the overall P-NP comparison. All women were included in these analyses and the patterns of expression were examined using clustering analysis in women classified according to TSLP (< 5, 5–10, or > 10 years). Considering upregulated and downregulated genes separately, K-means cluster analyses were performed using Multiexperiment Viewer software (MeV- v.4.8.1) [15], with Pearson uncentered as distance metric. We examined the clusters formed after randomly setting the number of clusters (K) to 2, 3, 4, or 5.

### Mining for functional categories and pathways

Data mining methods were applied to the differentially expressed genes to detect biological processes and pathways affected by parity. Ingenuity® Pathways Analysis (IPA) software version 24390178 (QIAGEN) was used to investigate canonical pathways. Gene Ontology (GO) enrichment analysis was performed using conditional hypergeometric tests in the Bioconductor GOstats package [16]. We carried out analyses separately for each cluster of upregulated genes. GO (gene ontology) terms with *p* value < 0.01 were considered enriched. To evaluate the GO terms enriched by each cluster of genes, the terms were grouped into broader terms (developmental process, immune response, or others) following the GO hierarchical tree graph view from GO consortium [17]. Few genes were identified as downregulated; therefore, we did not conduct GO analyses for these genes but rather examined the literature to identify their roles.

In addition, genes that were validated by real-time RT-PCR were used for analysis into cBioPortal for Cancer Genomics (http://www.cbioportal.org/) [18, 19]. We evaluated whether these genes have been described to be modified in breast cancer cases available in the cBioPortal databank; in addition, an overall survival Kaplan-Meier curve was generated. In total, 11 genes were evaluated among 5796 breast cancer patients.

### Validation through real-time RT-PCR

Eleven genes were selected for real-time RT-PCR analyses based on their biological roles in cell differentiation, proliferation, and chromatin remodeling. The assays were performed in the subset of 17 NP and 20 P samples (10 with TSLP < 5, and 10 with TSLP > 5) from whom sufficient RNA remained available, using TaqMan® Gene Expression Master Mix and TaqMan® Gene Expression Assays (Life Technologies). The end point used in the RT-PCR quantification, Ct, was defined as the PCR cycle number at which each assay target passes the threshold. Each assay was normalized to 18S, a housekeeping gene used as endogenous control (ΔCt). The difference between parous and nulliparous were estimated as the difference in mean ΔCt values (−ΔΔCt). To assess the statistical significance of the differences between P and NP, batch-adjusted *p* values were calculated using linear regression and comparisons with *p* value < 0.10 and fold change of at least 1.2 were considered statistically significant. Gene expression measured using RT-PCR and Affymetrix arrays were compared in the 37 subjects for whom both assays were used. Fold changes were estimated from multiple regressions using batch-adjusted, RMA (Robust Multi-Array Average)-normalized gene expression intensities, and intraclass and Spearman correlation coefficients were calculated.

### Immunohistochemical (IHC) staining

Paraffin sections at 4 μm were deparaffinized and placed in the antigen unmasking solution (Vector Laboratories, Burlingame, CA) and microwaved for 10 min at 100 °C. After cooling for 20 min, slides were quenched with Peroxide Block (BioGenex, Fremont, CA; #HK111) for 10 min, followed by blocking with Power Block (BioGenex, #HK085) for 20 min at room temperature. The sections were then stained with primary antibodies using a i6000 BioGenex Autostainer following standard protocol. The antibodies used were as follows: Cytokeratin 5 (BioGenex, #AN484-10 M, pre-diluted), CD123 (BD Biosciences, #554527; 1:400 dilution), LAMP3 (Abcam, #ab111090), Desmocollin 3 (Abcam, #ab190118; 1:150 dilution), CD2 (Abcam, #ab131276; 1:200 dilution), and CD3D (Abcam, #ab109531; 1:150 dilution). A Super Sensitive TM Polymer-HRP Detection System (BioGenex; #QD430-XAKE) was used to detect the staining. The images were acquired at × 400 magnification using an Aperio Digital Pathology Slide Scanner (Leica Biosystems, Buffalo Grove, IL) and analyzed by Aperio ImageScope Software (Leica Biosystems).

### Laser capture microdissection (LCM)

In additional samples, we conducted a substudy to assess how gene expression in RNA extracted from whole breast tissue relates to gene expression in RNA extracted from epithelial and stromal tissues. Breast biopsy tissue fixed in RNA later was frozen and cryostat was used for histological sectioning. The frozen sections were then stained with hematoxylin and eosin specially prepared utilizing RNAse-free water to avoid RNA degradation [20]. LCM was performed using the VERITAS Microdissection Instrument (Arcturus, CA, USA) to select and capture all the epithelial tissue present in each section. The tissue left on the slide was then scrapped into a different tube and classified as stroma. The RNA extraction from the collected cells was performed using the Arcturus® PicoPure®RNA Isolation Kit (Life Technologies). RNA was also extracted from a second core biopsy, in which no LCM was performed (called hereafter whole tissue, WT).

For each woman included in this substudy, three microarrays were performed in the same batch, using RNA extracted from (1) the epithelial cells of the mammary gland, (2) the stroma, and (3) WT. RNA amplification and labelling was performed using MessageAmpTM Premier RNA Amplification Kit (Life Technologies), and the arrays were hybridized to Affymetrix Human Genome Gene Chip U133 Plus 2.0 arrays. All arrays were subjected to QC analysis as described earlier. The arrays were RMA pre-processed, and probesets with < 75% of present calls and/or low CV (i.e., CV in the first quartile) were filtered out, resulting in 10,252 probesets for analysis. All values were log-transformed and normalized prior to analysis.

Nine subjects (5 NP and 4 P) had successful arrays for whole tissue, epithelial tissue, and stromal tissue. For each subject, a linear regression model was fitted across all genes. The gene expression values in the whole tissue were modeled as a linear function of the gene expression in epithelial and stromal tissues.

Gene expression comparison between the breast tissue types was performed for ten subjects (5 P and 5 NP) with successful epithelium and stroma arrays. For each probe, the fold changes were calculated as the median of within subject fold changes [expression in epithelium] / [expression in stroma] for each subject. A paired two-sample *t*-test was performed for each probe set, and *p* values were adjusted for multiple comparisons using the FDR approach. Probes with FDR < 10% and fold changes of at least 20% were considered statistically different between epithelium and stroma. GO analysis was performed using the same methodology described above. The small number of samples limited our gene expression analyses between tissue types in function of parity.

## Results

### Volunteers included in the analysis

**Fig. 1:**
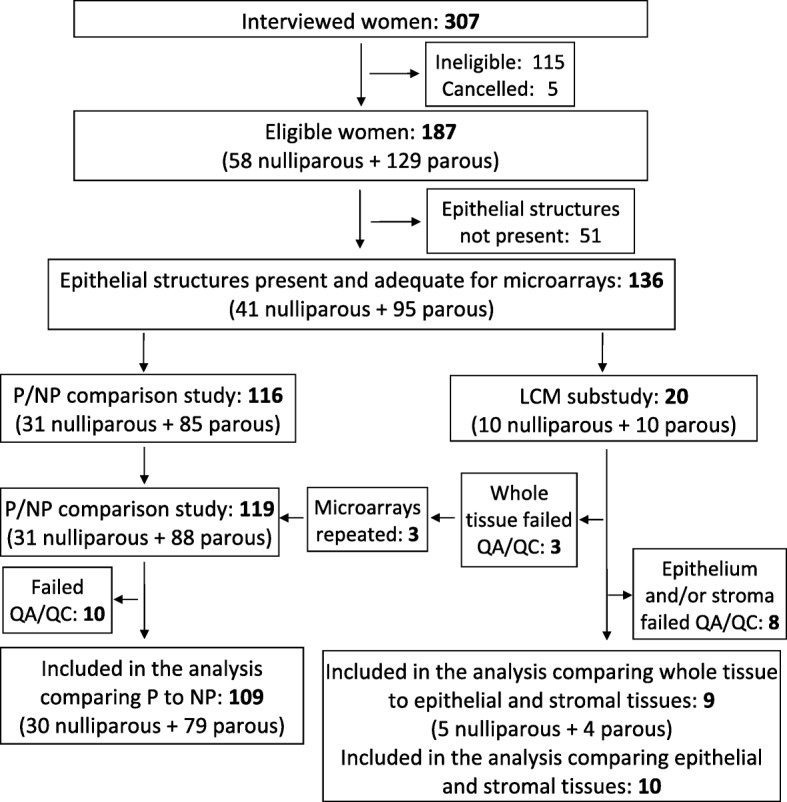
Subject accrual and sample processing summary. Figure shows the number of women who volunteered, reasons for exclusion, and allocation of samples to the P/NP comparison study and LCM substudy

A total of 307 women between ages 29 and 47 volunteered between March 2011 and June 2012 (Fig. 1). After exclusions related to eligibility, lack of epithelial structures, or QC failures, samples from 109 women (30 NP and 79 P) were included in the main study comparing P vs. NP, and samples from 10  women were included in the LCM substudy comparing the tissue types (Fig. 1).

P and NP women were similar with respect to most characteristics, such as age of menarche, breast density, and body mass index (BMI) (Table 1). However, the median age and median smoking duration were lower in NP than P women, and a larger proportion of P had a hormonal intra-uterine device (IUD).

**Table 1:** Descriptive characteristics of the study subjects

Characteristics	Parous (*n* = 79)	Nulliparous (*n* = 30)	*p* value
Age at visit, years	39.9 (30.1, 47.3)	35.8 (30.1, 46.2)	0.03
Number of FTP			
1	13 (16%)		
2	44 (46%)		
3+	22 (28%)		
Time since last FTP, years			
≤ 5	30 (39%)		
6–10	29 (37%)		
> 10	20 (25%)		
Age at first FTP, years	26 (18, 27)		
Phase of cycle/hormonal IUD^1^			0.05
Luteal	21 (28%)	14 (52%)	
Ovulatory	10 (13%)	4 (15%)	
Follicular	21 (28%)	7 (26%)	
Hormonal IUD	23 (31%)	2 (7%)	
Missing	4	3	
OC ever use	74 (94%)	27 (90%)	0.68
Breast density, BIRADS			
1	10 (13%)	4 (13%)	0.38
2	16 (20%)	2 (7%)	
3	10 (13%)	4 (13%)	
4	43 (54%)	20 (67%)	
Age at menarche, years	13.0 (11.0, 16.0)	13.0 (11.0, 15.0)	0.60
Family history of breast cancer	7 (9%)	2 (7%)	0.99
Height, cm	165.0 (148.0, 184.0)	166.5 (157.0, 174.0)	0.77
Weight, kg	68.0 (41.0, 115.0)	64.0 (46.0, 97.0)	0.38
BMI	24.2 (18.7, 38.0)	23.4 (18.7, 37.1)	0.24
Smoking			
Never	51 (64%)	20 (67%)	0.15
Past	23 (29%)	5 (17%)	
Current	5 (6%)	5 (17%)	
Smoking duration, years	8.0 (0.5, 20.0)	17.0 (7.0, 30.0)	0.006
Years since quitting (past smokers)	13.0 (0.1, 26.0)	6.0 (1.0, 12.0)	0.09

^1^*p* value for IUD/ no IUD = 0.01; excluding women having an IUD, there was no statistically significant difference in phase of menstrual cycle between P and NP

### Differential gene expression

Using the discovery and validation approach described in “Methods”, 54 probesets, representing 43 genes (Table 2), were differentially expressed between P and NP women. Of the 43 genes, 40 were upregulated and 3 downregulated in the parous premenopausal breast (Table 2). Upregulated genes in the P breast included APOBEC3G, DSC3, FZD8, and EAF2, while FOXQ1 was among the downregulated genes.

**Table 2:** Genes differentially expressed between parous and nulliparous premenopausal women (discovery/validation approach)

ProbeID	EntrezID	Symbol	GeneName	FDR	Fold change
Genes upregulated in parous women					
206641_at	608	TNFRSF17	Tumor necrosis factor receptor superfamily, member 17	0.013	2.41
228504_at	6332	SCN7A	Sodium channel, voltage-gated, type VII, alpha subunit	0.006	2.15
237625_s_at	3514	IGKC	Immunoglobulin kappa constant	0.006	1.94
222838_at	57823	SLAMF7	SLAM family member 7	0.017	1.93
224342_x_at	96610	LOC96610	BMS1 homolog, ribosome assembly protein (yeast) pseudogene	0.032	1.80
206121_at	270	AMPD1	Adenosine monophosphate deaminase 1	0.021	1.76
206033_s_at	1825	DSC3	Desmocollin 3	0.006	1.68
1555759_a_at	6352	CCL5	Chemokine (C-C motif) ligand 5	0.018	1.67
206478_at	9834	KIAA0125	KIAA0125	0.006	1.65
213193_x_at	28639	TRBC1	T cell receptor beta constant 1	0.006	1.60
207651_at	29909	GPR171	G protein-coupled receptor 171	0.014	1.60
206310_at	6691	SPINK2	Serine peptidase inhibitor, Kazal type 2 (acrosin-trypsin inhibitor)	0.006	1.59
231647_s_at	83416	FCRL5	Fc receptor-like 5	0.012	1.59
203130_s_at	3800	KIF5C	Kinesin family member 5C	0.023	1.58
205831_at	914	CD2	CD2 molecule	0.012	1.54
216430_x_at	28823	IGLV1-44	Immunoglobulin lambda variable 1-44	0.034	1.52
211339_s_at	3702	ITK	IL2-inducible T cell kinase	0.016	1.50
206666_at	3003	GZMK	Granzyme K (granzyme 3; tryptase II)	0.030	1.49
204562_at	3662	IRF4	Interferon regulatory factor 4	0.017	1.47
213539_at	915	CD3D	CD3d molecule, delta (CD3-TCR complex)	0.011	1.45
206181_at	6504	SLAMF1	Signaling lymphocytic activation molecule family member 1	0.014	1.45
206150_at	939	CD27	CD27 molecule	0.019	1.44
206991_s_at	1234	CCR5	Chemokine (C-C motif) receptor 5 (gene/pseudogene)	0.010	1.41
235153_at	138065	RNF183	Ring finger protein 183	0.026	1.41
214470_at	3820	KLRB1	Killer cell lectin-like receptor subfamily B, member 1	0.038	1.40
230011_at	150365	MEI1	Meiosis inhibitor 1	0.010	1.39
211902_x_at	10730	YME1L1	YME1-like 1 (*S. cerevisiae*)	0.015	1.37
221584_s_at	3778	KCNMA1	Potassium large conductance calcium-activated channel, subfamily M, alpha member 1	0.034	1.36
219551_at	55840	EAF2	ELL associated factor 2	0.015	1.35
220402_at	63970	TP53AIP1	Tumor protein p53 regulated apoptosis inducing protein 1	0.015	1.35
206761_at	10225	CD96	CD96 molecule	0.036	1.33
212314_at	23231	SEL1L3	Sel-1 suppressor of lin-12-like 3 (*C. elegans*)	0.014	1.31
204682_at	4053	LTBP2	Latent transforming growth factor beta binding protein 2	0.006	1.31
207655_s_at	29760	BLNK	B cell linker	0.015	1.31
204205_at	60489	APOBEC3G	Apolipoprotein B mRNA-editing enzyme, catalytic polypeptide-like 3G	0.014	1.26
223322_at	83593	RASSF5	Ras association (RalGDS/AF-6) domain family member 5	0.024	1.25
227405_s_at	8325	FZD8	Frizzled family receptor 8	0.024	1.24
211469_s_at	10663	CXCR6	Chemokine (C-X-C motif) receptor 6	0.019	1.22
233555_s_at	55959	SULF2	Sulfatase 2	0.032	1.20
209574_s_at	753	LDLRAD4	Low-density lipoprotein receptor class A domain containing 4	0.045	1.15
Genes downregulated in parous women					
227475_at	94234	FOXQ1	Forkhead box Q1	0.026	0.64
244680_at	2743	GLRB	Glycine receptor, beta	0.046	0.79
236399_at	54103	PION	Pigeon homolog (Drosophila)	0.041	0.86

**Fig. 2:**
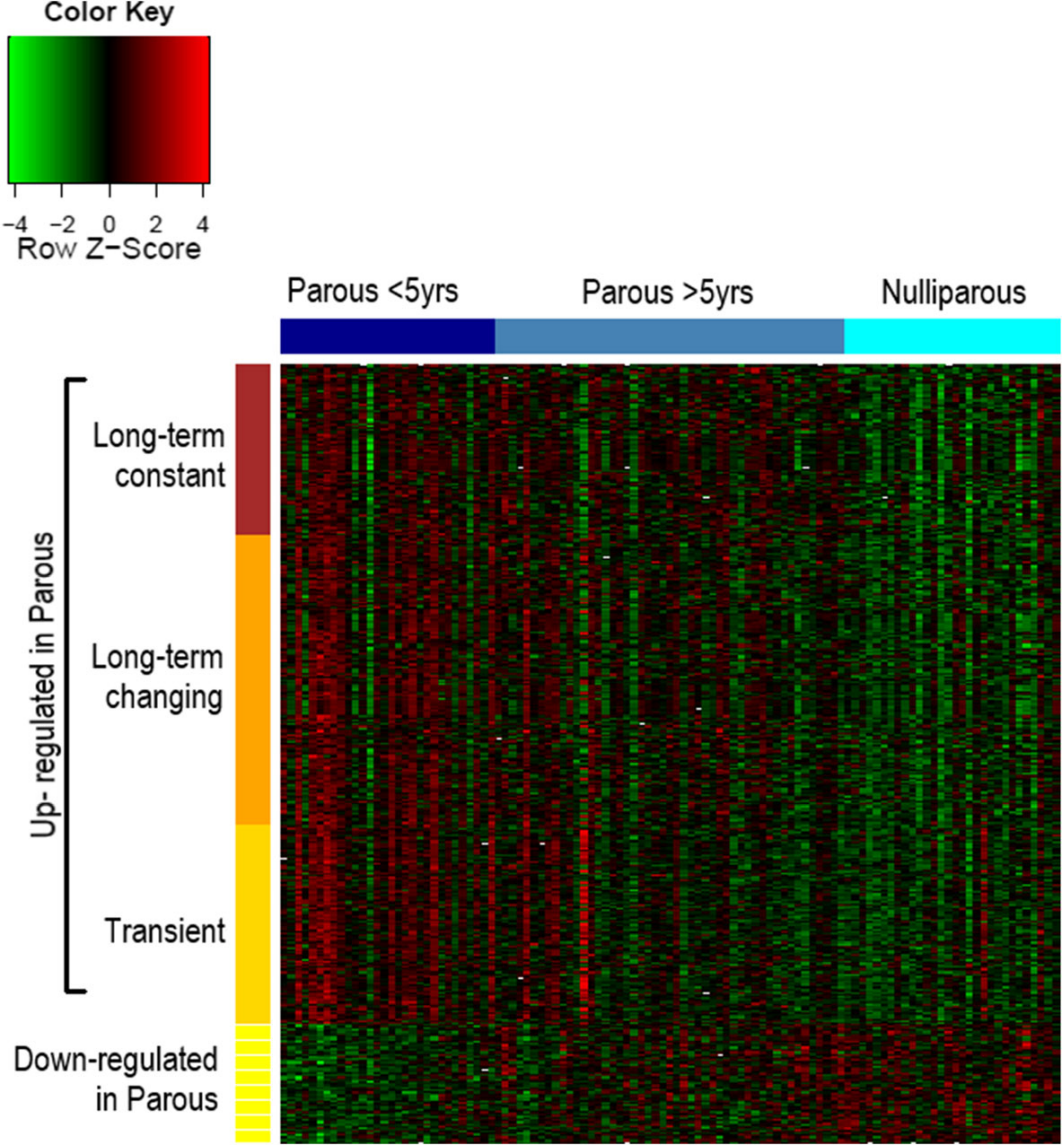
Heatmap of all 416 probesets found differentially expressed between parous and nulliparous. Each column represents a volunteer. Dark blue represents parous women within 5 years since last full-term pregnancy, blue represents parous women with more than 5 years since pregnancy, and cyan represents nulliparous women. Each line corresponds to a probeset, which accordingly with its pattern of expression within the parous breast samples compared to nulliparous, was classified as downregulated (light yellow), transiently upregulated (yellow), long-term changing (orange), and long-term constant upregulated (brown)

Analyses of gene expression according to TSLP identified 286 genes (416 probesets) differentially expressed between P and NP samples (Fig. 2). Among these, 238 genes (352 probesets) were upregulated in P women, while 48 genes (64 probesets) were downregulated (Additional file 1).

**Fig. 3:**
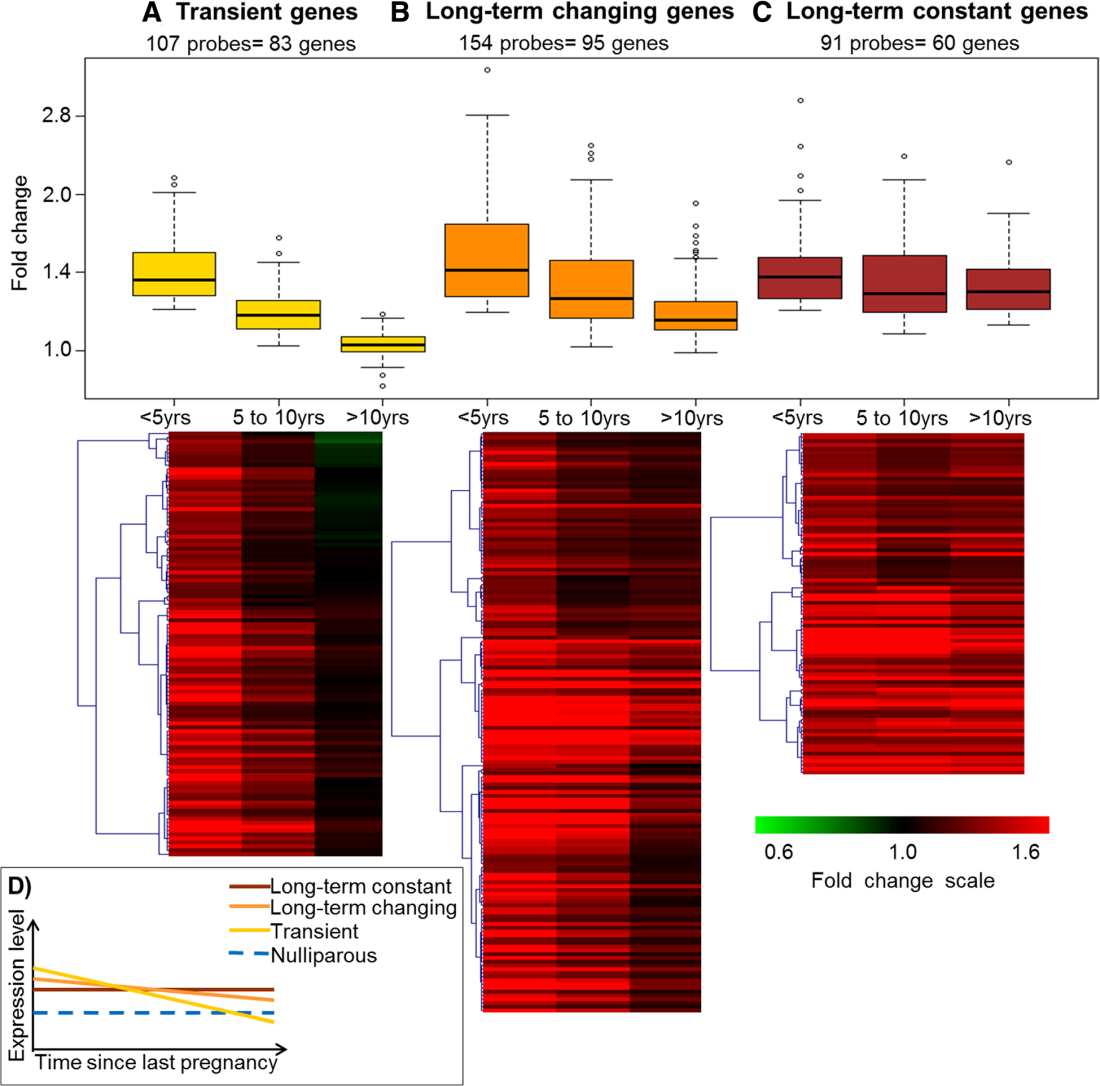
Three expression patterns were identified among the upregulated genes. In the top, the graph shows the fold change ranges of the each cluster: **a** transient, **b** long-term changing, and **c** long-term constant according to time since last full-term pregnancy (< 5 years, 5–10 years, and > 10 years). Heatmaps show the expression patterns of each cluster. **d** Schematic representation of the expression levels of each cluster in function of time since last pregnancy

For probesets upregulated in the P women, gene expression differences clustered in three expression patterns as a function of TSLP (Fig. 3). The first cluster consisted of 83 genes (107 probesets) which were upregulated following the last FTP, but whose expression progressively returned to the level of expression observed in NP women (Fig. 3a). These 83 genes were named “transient” genes. The second cluster consisted of probesets for which the P-NP differences were the highest for women with < 5 years since last FTP, decreased with increasing TSLP but remained elevated as compared to NP women. The 95 genes (154 probesets) in this cluster were called “long-term changing” (Fig. 3b). In the last cluster, which included 60 genes (91 probesets), the fold changes between P an NP appeared constant, regardless of the TSLP. Therefore, we called these “long-term constant” genes (Fig. 3c).

**Fig. 4:**
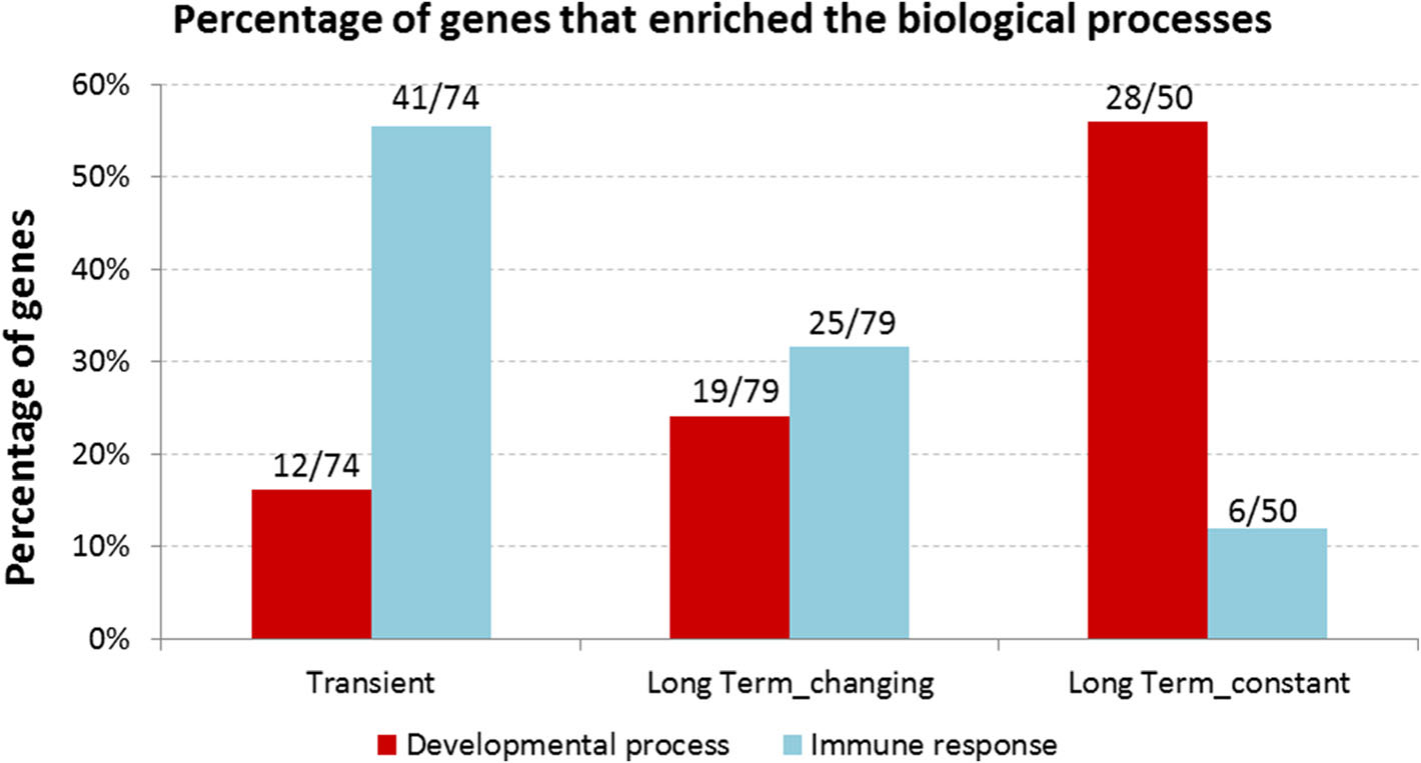
Graph shows the percentage of genes associated with GO terms related to developmental processes (red) and immune response (blue) for each of the three clusters. 74, 79, and 50 are the total number of genes associated at least with one GO term for each cluster

Gene Ontology (GO) enrichment analyses showed an abundance of GO terms associated to developmental processes or immune response (Fig. 4), other less-abundant enriched GO terms were related to proliferation, intracellular transport, and cell death. Among the genes whose expression was transiently affected by parity 55%, including CD8A, XCL1, and GZMA (Table 3), enriched GO terms related to immune response (Additional file 2). Among the genes classified as long-term changing, 32% were related to immune response (e.g., CD2) and 24% were involved in developmental processes (e.g., EAF2) (Table 3, Additional file 3). Notably, of the long-term constant genes, 56% were related to developmental/differentiation processes (Additional file 4), including EGR3, DSC3, KRT5, and FZD8 (Table 3). These data indicate that the proportion of genes involved in immune response decreases among the genes that are upregulated irrespectively of TSLP, while the proportion of genes related to developmental processes increases (chi-squared *p* value = *p* < 0.001).

**Table 3:** Genes that enriched developmental processes and immune response gene ontologies

Transient genes					
Developmental process					
*ANXA1*	*CAMK4*	*CD8A*	*CD27*	*EOMES*	*ITK*
*JAK3*	*LCK*	*PTPN22*	*PTPRC*	*SASH3*	*THEMIS*
Immune response					
*ANXA1*	*CAMK4*	CCL5	*CD8A*	*CD27*	CD48
CD96	CD247	CORO1A	CST7	CXCL14	CXCL9
*EOMES*	FASLG	FYB	GZMA	HCST	IGLC1
IKZF1	IL16	IL7R	ITGAL	*ITK*	*JAK3*
KLHL6	LAT	LAX1	*LCK*	LCP2	LY9
PRKCB	*PTPN22*	*PTPRC*	*SASH3*	SELPLG	SEMA4D
sSH2D1A	SLAMF1	*THEMIS*	TRAC	XCL1	
Long-term changing genes					
Developmental process					
*C3*	*CCR2*	*CD2*	*CXCL10*	DACT1	DKK3
EAF2	EPHA7	FGF1	FGFR2	HCLS1	*HLA-DOA*
*IL12RB1*	*IRF4*	LAMA2	OSR2	PDGFRA	SIPA1L1
SPHK1					
Immune response					
APOBEC3G	BLNK	C1S	*C3*	*CCR2*	*CD2*
CD3D	CD38	CRTAM	*CXCL10*	GPR183	*HLA-DOA*
HLA-DPB1	IGKC	*IL12RB1*	*IRF4*	LPXN	MZB1
NFATC2	PAWR	POU2AF1	PRKCQ	SAMSN1	SLAMF7
TRBC1					
Long-term constant genes					
Developmental process					
BHLHE22	*CCL19*	*CCL2*	DCN	DSC3	EGR3
EPHB1	FHL2	FZD8	*GLI3*	GPR65	KCNMA1
KIF5C	KRT5	MYLK	NFASC	PRKCA	SALL1
SDC1	SULF1	SULF2	TAGLN	TREM2	TYMS
*VCAM1*	WIPF3	XDH	ZIC1		
Immune response					
*CCL2*	*CCL19*	*GLI3*	HLA-DPA1	HLA-DRA	*VCAM1*

Italicized genes enriched both developmental and immune response processes

**Fig. 5:**
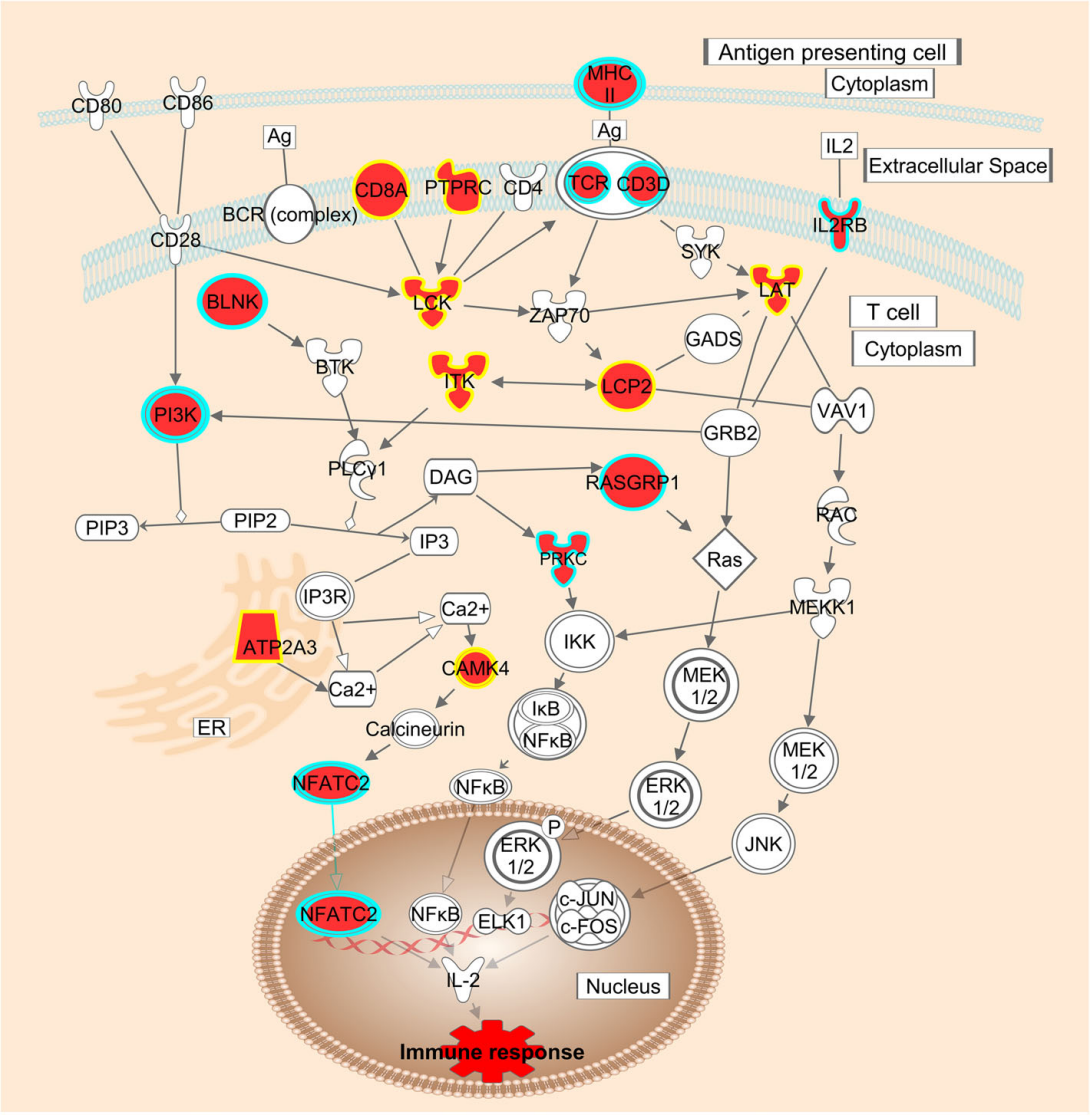
Pathway representing T cell signaling. Genes in red were upregulated in the parous breast. Genes with a blue border had a long-term effect, while genes with a yellow border were transiently upregulated. Pathway was modified from IPA software (QIAGEN) using Path Designer

Evaluation of the canonical pathways represented by the differentially expressed genes also showed that pathways involved in signaling and activation of T cells were enriched by both transient and long-term genes (Fig. 5).

The 48 genes (64 probesets) downregulated in the P breast (Table 4) fell into two patterns. Twenty-two genes, including WIF1, EDN1, CXCL1, and FOXQ1, were downregulated in the P breast and remained with lower expression levels compared to NP breast irrespective of the number of years since last FTP. The remaining 26 genes were downregulated in women with TSLP < 5 years; however, the expression increased reaching similar or higher levels than those in NP women when TSLP increased. These genes included DLG5, KDM4B, and TOX3. We did not conduct a GO enrichment analysis for these genes because of their limited number.

**Table 4:** Genes downregulated within parous breast

EntrezID	ProbeID	Symbol	GeneName
Genes downregulated constantly			
653	205431_s_at	BMP5	Bone morphogenetic protein 5
694	1559975_at	BTG1	B cell translocation gene 1, anti-proliferative
285382	242447_at	C3orf70	Chromosome 3 open reading frame 70
1277	202310_s_at	COL1A1	Collagen, type I, alpha 1
131873	242641_at	COL6A6	Collagen, type VI, alpha 6
2919	204470_at	CXCL1	Chemokine (C-X-C motif) ligand 1 (melanoma growth-stimulating activity, alpha)
55184	219951_s_at	DZANK1	Double zinc ribbon and ankyrin repeat domains 1
1906	222802_at	EDN1	Endothelin 1
133121	229916_at	ENPP6	Ectonucleotide pyrophosphatase/phosphodiesterase 6
94234	227475_at	FOXQ1	Forkhead box Q1
2743	205280_at	GLRB	Glycine receptor, beta
253012	242601_at	HEPACAM2	HEPACAM family member 2
3736	230849_at	KCNA1	Potassium voltage-gated channel, shaker-related subfamily, member 1 (episodic ataxia with myokymia)
79442	219949_at	LRRC2	Leucine-rich repeat containing 2
9848	205442_at	MFAP3L	Microfibrillar-associated protein 3-like
4300	204918_s_at	MLLT3	Myeloid/lymphoid or mixed-lineage leukemia (trithorax homolog, Drosophila); translocated to, 3
3340	1554010_at	NDST1	*N*-Deacetylase/*N*-sulfotransferase (heparan glucosaminyl) 1
11069	205651_x_at	RAPGEF4	Rap guanine nucleotide exchange factor (GEF) 4
950	201647_s_at	SCARB2	Scavenger receptor class B, member 2
1317	236217_at	SLC31A1	Solute carrier family 31 (copper transporters), member 1
116441	228489_at	TM4SF18	Transmembrane 4 L six family member 18
11197	204712_at	WIF1	WNT inhibitory factor 1
Genes downregulated transiently			
170692	230040_at	ADAMTS18	ADAM metallopeptidase with thrombospondin type 1 motif, 18
267	202203_s_at	AMFR	Autocrine motility factor receptor, E3 ubiquitin protein ligase
56892	218541_s_at	C8orf4	Chromosome 8 open reading frame 4
401546	229964_at	C9orf152	Chromosome 9 open reading frame 152
760	209301_at	CA2	Carbonic anhydrase II
54102	227742_at	CLIC6	Chloride intracellular channel 6
1281	211161_s_at	COL3A1	Collagen, type III, alpha 1
9231	229689_s_at	DLG5	Discs, large homolog 5 (Drosophila)
54898	213712_at	ELOVL2	ELOVL fatty acid elongase 2
2069	205767_at	EREG	Epiregulin
2330	205776_at	FMO5	Flavin containing monooxygenase 5
2717	214430_at	GLA	Galactosidase, alpha
26585	218469_at	GREM1	Gremlin 1, DAN family BMP antagonist
2892	230144_at	GRIA3	Glutamate receptor, ionotropic, AMPA 3
23704	222379_at	KCNE4	Potassium voltage-gated channel, Isk-related family, member 4
23030	212492_s_at	KDM4B	Lysine (K)-specific demethylase 4B
5366	204286_s_at	PMAIP1	Phorbol-12-myristate-13-acetate-induced protein 1
11098	202458_at	PRSS23	Protease, serine, 23
5744	211756_at	PTHLH	Parathyroid hormone-like hormone
157869	214725_at	SBSPON	Somatomedin B and thrombospondin, type 1 domain containing
1811	206143_at	SLC26A3	Solute carrier family 26, member 3
51012	229835_s_at	SLMO2	Slowmo homolog 2 (Drosophila)
6431	206108_s_at	SRSF6	Serine/arginine-rich splicing factor 6
27324	216623_x_at	TOX3	TOX high mobility group box family member 3
7366	207392_x_at	UGT2B15	UDP glucuronosyltransferase 2 family, polypeptide B15
151126	1555801_s_at	ZNF385B	Zinc finger protein 385B

Finally, we examined genes reported to be related to chromatin remodeling. Among those upregulated, we observed APOBEC3G, TOX, UHRF1, and NAP1L2, while KDM4B and TOX3 were downregulated.

### Validation of microarray results

P/NP differences in gene expression were confirmed for eight out of the 11 genes analyzed by real-time PCR in a subset of 37 samples (20 P and 17 NP); Ct values, ∆Cts, and −∆∆Cts are shown in Additional file 5. WIF1 was downregulated, while EAF2, BHLHE22, APOBEC3G, DSC3, KRT5, EGR3, and RASGRP1 were upregulated in the P breast (Table 5). The intraclass correlation coefficient (ICC) comparing the real-time to microarray measurements varied from 0.35 (EAF2) to 0.92 (WIF1), and Spearman correlation coefficients varied from 0.41 (EAF2) to 0.94 (WIF1) (Table 5).

**Table 5:** Correlation of real-time RT-PCR and microarray results

Genes	Affymetrix probeset	TaqMan Assay ID	Microarray		Real time		ICC	Spearman correlation
			FC	P	FC	P		
WIF1	204712_at	Hs00183662_m1	0.39	0.055	0.32	0.034	0.92	0.94
FOXQ1	227475_at	Hs00536425_s1	0.64	0.020	0.70	0.218	0.64	0.69
FZD8	227405_s_at	Hs00259040_s1	1.3	0.003	1.17	0.188	0.43	0.42
SDC1	201286_at	Hs00896423_m1	1.48	0.014	1.19	0.456	0.73	0.62
EAF2	219551_at	Hs00218407_m1	1.36	0.008	1.24	0.059	0.35	0.41
BHLHE22	228636_at	Hs01084964_s1	1.24	0.078	1.35	0.022	0.41	0.53
APOBEC3G	204205_at	Hs00222415_m1	1.44	0.000	1.44	0.001	0.39	0.48
DSC3	206033_s_at	Hs00170032_m1	1.70	0.002	1.45	0.063	0.64	0.51
KRT5	201820_at	Hs00361185_m1	1.51	0.012	1.54	0.062	0.64	0.71
EGR3	206115_at	Hs00231780_m1	1.71	0.026	1.57	0.072	0.76	0.70
RASGRP1	205590_at	Hs00996727_m1	1.61	0.002	1.71	0.002	0.66	0.74

Correlation of real-time RT-PCR and microarray results were calculated using the 20 parous vs. 17 nulliparous samples, for whom enough RNA material was still available. *FC* mean fold change, *P* batch-adjusted *p* value, *ICC* intraclass correlation coefficient

**Fig. 6:**
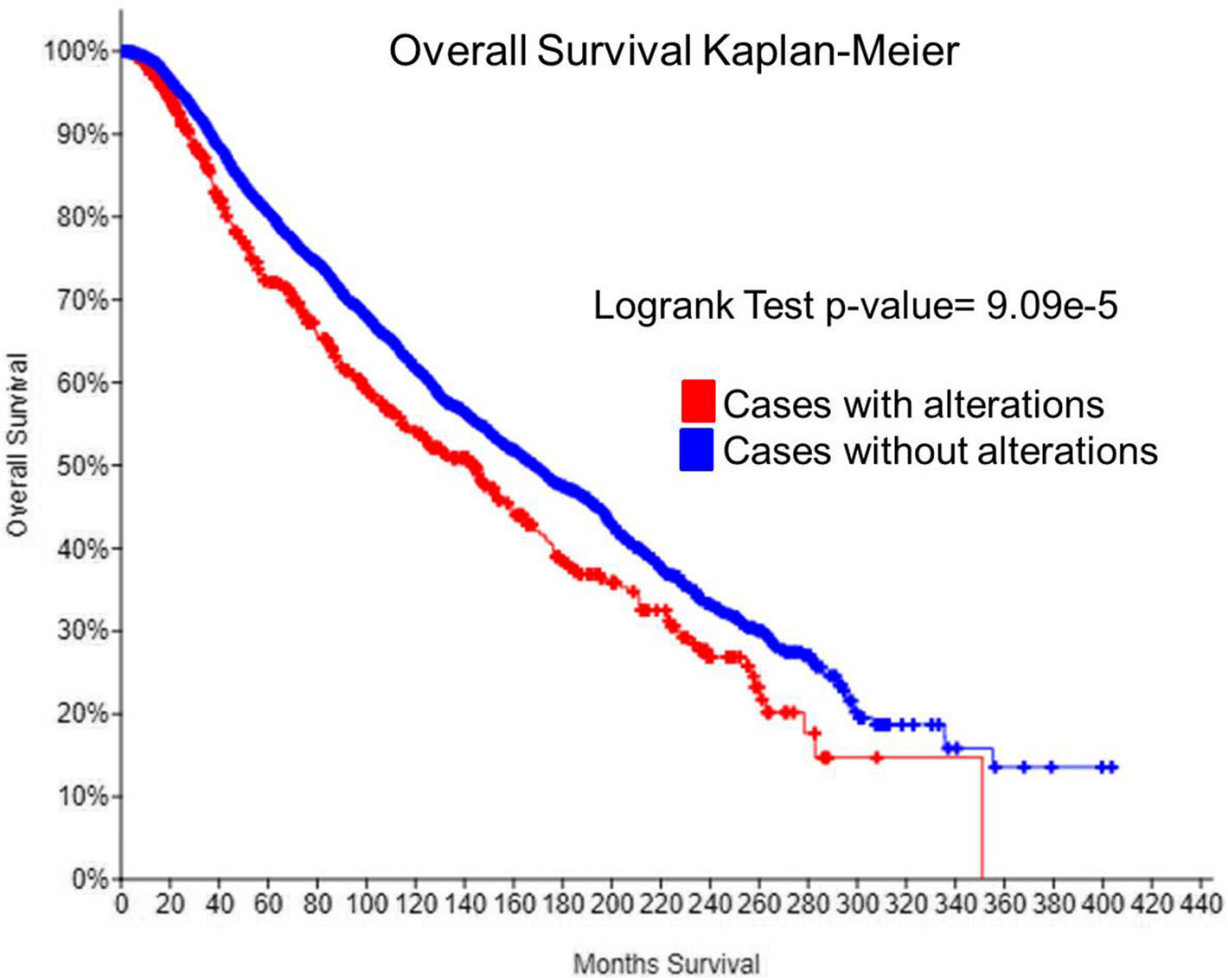
Analyses of the parous differentially expressed genes in breast cancer patients. Genes that were selected for real-time RT-PCR validation were also used for evaluation into breast cancer patients using the cBioPortal tool (http://www.cbioportal.org/), overall Kaplan-Meier survival curve comparing between the cases with and without alterations in these genes. Patients that contained alterations had a median of 143.13 months overall survival while patients without alteration in these genes had median of 168.3 (logrank test *p* value = 9.09e−5)

We also analyzed the 11 selected genes in 5796 patients of breast cancer available in cBioPortal. One or more of these genes were altered in 770 (13%) breast cancer patients. BHLHE22 was the gene that appears altered in the largest percentage of patients (6.9%); the alterations included amplification, deep deletion, and missense mutations. The other ten genes showed to be altered in 1.5% or less patients (Table 6). An overall Kaplan-Meier survival curve has been generated comparing the group of patients with these 11 genes altered versus patients without the alterations. The group of patients that contain alteration in these genes has shorter overall survival, median of 143.13 versus 168.3 months, logrank test *p* value = 9.09e−5 (Fig. 6).

**Table 6:** Gene alteration in 5796 breast cancer subjects

Genes	Number of samples altered	Percent of samples altered
BHLHE22	400	6.9
EGR3	87	1.5
WIF1	73	1.3
FOXQ1	73	1.3
FZD8	64	1.1
DSC3	57	1.0
KRT5	55	1.0
SDC1	35	0.6
EAF2	30	0.5
RASGRP1	30	0.5
APOBEC3G	28	0.5

**Fig. 7:**
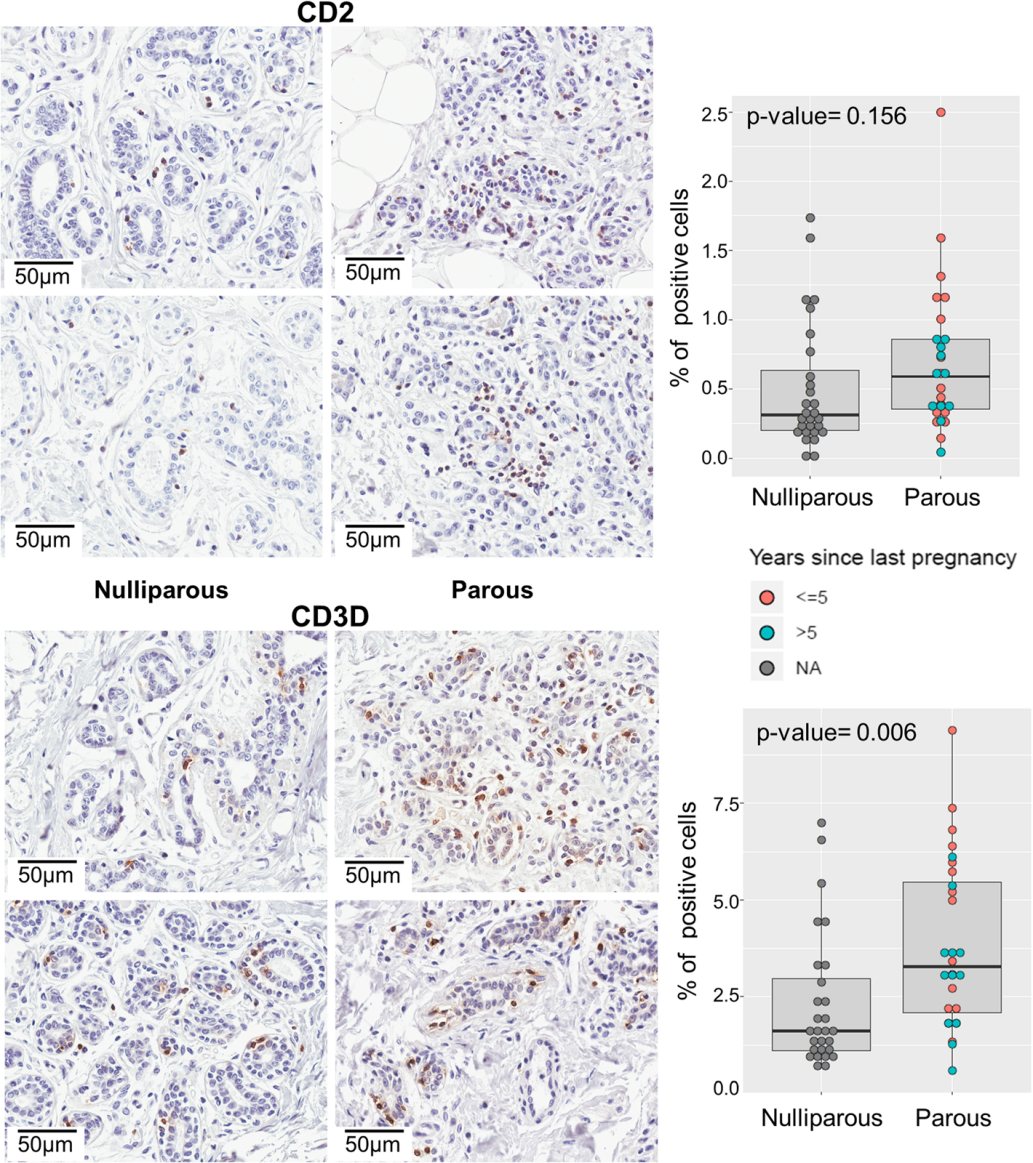
Immunohistochemical evaluation of CD2 and CD3D for T cell activation. On the top are results regarding CD2, and on the bottom are results of CD3D. Panels on the left show the imunohistochemical reactions (× 400), each panel contains two nulliparous samples (left) and two parous samples (right). On the right, boxplots show  the percentage of positive cells. We observe an increase in the percentage of CD3D-positive cells in the parous group (*p* value = 0.006)

We performed IHC analyses to evaluate whether the changes in gene expression reflected into the protein expression levels. We have evaluated two proteins related to differentiation Desmocollin 3 and Cytokeratin 5. These two markers were presented in all tested samples, and although we have observed slight upregulation of their genes by microarray and RT-PCR, we did not detect protein differences between P and NP breast tissues. Related to immune response, we evaluated markers for dendritic cells because of their role in antigen presentation for T cell activation. Using two markers CD123 and LAMP3, we detected few positive cells with no differences between P and NP samples. We also evaluated CD2 and CD3D, markers of T cell activation (Fig. 7). The percentage of positive cells for CD2 was low in both groups, we observed slight increase in the percentage of CD2-positive cells in parous women (1.6 times; P median = 0.59%, NP median = 0.31%); however, it was not statistically significant (*p* value = 0.156). The overall percentage of CD3D-positive cells was higher ranging from 0.6 to 9.4%. We confirmed a statistically significant twofold increase in the percentage of CD3D-positive cells in the P breast (P median = 3.28%, NP median = 1.62%, *p* value = 0.006) (Fig. 7). We also compared whether the percentage of positive cells between P women with TSLP ≤ 5 and > 5 differed. For both CD2 and CD3D, we see a slight larger number of positive cells in TSLP ≤ 5, but were not statistically significant (CD2 *p* value = 0.16 and CD3D *p* value = 0.09).

### Laser capture microdissection (LCM) results

We found that a linear function of gene expression levels in RNA from epithelial tissue and RNA from stromal tissue predicted extremely well the level of expression in RNA extracted from the whole tissue (0.91 < *R*^2^ < 0.95), i.e., the gene expression in whole tissue was a weighted average of the gene expression in epithelial tissue and in stromal tissue for all nine individuals included in this substudy (Table 7). This was observed regardless of the proportion of each tissue type found on the paraffin sections, which varied across individuals (regression coefficient for epithelial tissue ranged from 0.09 to 0.68).

**Table 7:** Linear regression coefficients of gene expression levels in breast tissue

Subject	β coefficients		*R* ^2^
	Epithelial tissue	Stromal tissue	
1	0.091	0.910	0.943
2	0.461	0.503	0.945
3	0.495	0.425	0.905
4	0.516	0.406	0.922
5	0.287	0.624	0.929
6	0.354	0.622	0.953
7	0.681	0.308	0.920
8	0.342	0.654	0.939
9	0.122	0.877	0.926

Analysis of gene expression differences between epithelium and stroma showed 730 genes (956 probesets) with higher expression levels in the epithelium, while 663 genes (1020 probesets) were more expressed in the stroma (Additional file 6). GO analyses of these genes demonstrated a broader range of biological processes enriched by the genes with higher expression levels in the stroma (306 GO terms), which included cell motility, cell signaling, angiogenesis, development, and lipid process among other processes. Conversely, the genes with higher expression in the epithelium, enriched 12 GO terms, consisting a biological process involved in epithelial development and tight junction among other GOs (Additional file 7).

## Discussion

This study evaluated gene expression differences in parous and non-parous breast using biopsies from healthy premenopausal volunteers. Using a discovery/validation approach, we found 43 differentially expressed genes (Table 2). Evaluation of expression differences between NP and P as a function of TSLP identified 238 genes up- and 48 genes downregulated in the P breast. The downregulated genes fell into two patterns of expression (transient and long-term), while the upregulated genes fell into three patterns (transient, long-term changing, and long-term constant) (Fig. 3). Through GO enrichment analyses, we found that genes whose expression was transiently increased after pregnancy were mainly related to immune response. Long-term changing genes included immune- and development-related genes, while genes categorized as long-term constant were mainly involved in cell differentiation and developmental processes (Fig. 4). LCM performed in a small set of samples indicated that the gene expression observed on whole-tissue arrays corresponded to the weighted average of the gene expression observed in the epithelial and stromal tissues.

Among the 43 differentially expressed genes (Table 2) found in our discovery/validation analysis were DSC3 and KRT5, whose differential expression was confirmed by RT-PCR. These genes were also found in our previous study conducted in postmenopausal women [6, 7], indicating that the expression of these genes is durably modified by pregnancy. Consistent with this observation, DSC3 and KRT5 fell into the “long-term constant” category in our analysis by TSLP. These two genes are involved in cell communication and epithelial differentiation [21, 22]. Additionally, DSC3 has been suggested to act as a tumor suppressor [23–26], and its silencing is a common event in breast tumors [26].

While a discovery/validation approach is valuable to reduce the chance of false-positive results, our sample size was fairly small, which can lead to unstable results and lack of detection of some associations [27, 28]. This was a concern particularly because the breast undergoes major remodeling after a pregnancy/breastfeeding. Thus, genes may go through successive changes in pattern of expression after pregnancy, and analyses ignoring time since last pregnancy could miss transient expression modifications. We therefore also examined gene expression differences according to time since last pregnancy. To the best our knowledge, this is the first study that used a whole-transcriptome approach to demonstrate a cluster of biological functions following distinct expression patterns in the human breast following pregnancy.

The observation that the “long-term constant” genes were mainly involved in cell differentiation and developmental processes (Fig. 4) is consistent with the transcriptomic profile we previously described in the parous postmenopausal breast, in which upregulated genes showed enrichment of similar processes [6–8]. These findings indicate that the parity signature set after pregnancy remains until the postmenopausal years. Other development-related genes upregulated in the P breast and confirmed by RT-PCR were RASGRP1 (RAS guanyl-releasing protein 1—calcium and DAG-regulated), EGR3 (early growth response 3), and BHLHE22 (basic helix-loop-helix family member e22). These genes, in addition to differentiation, are known to regulate proliferation and cell growth [29–32].

Among the genes classified as developmental, we also found components of the WNT pathway. WNT canonical and non-canonical pathways participate in cell fate determination, cell polarity, adhesion, and motility [33, 34], all important functions in the differentiation of the breast. Differentiation induced by parity has been demonstrated to alter WNT/Notch signaling in mice [35], and we have described alterations in the methylation profile of genes belonging to this pathway and its regulation in the postmenopausal breast [36]. In the current study, we observed two genes of this pathway upregulated in the P breast, FZD8 (frizzled family receptor 8) which is a transmembrane receptor transducing WNT signals, and EAF2 (ELL-associated factor 2), which is important for early embryonic development and critical for adult tissue homeostasis and prevention against tumor initiation [37, 38]. WNT inhibitory factor 1, WIF1, was downregulated in P women, and although methylation of WIF1 has been observed in several tumors [39], including breast cancer [40], this can be an indication that the WNT pathway has an important role in the shifting of the stem cells to a more differentiated status in the P breast, as demonstrated earlier [8, 41]. Yet related to WNT pathway, we found FOXQ1 or forkhead box Q1, constantly downregulated in the P breast. This gene is a direct target of the canonical WNT pathway and its overexpression has been associated with different tumors and cancer cell lines [42]. Suppression of FOXQ1 inhibits cell proliferation, motility/invasion, and epithelial-mesenchymal transition phenotypes in cancer cells [43–45]; a similar effect could be predicted in the breast of P women.

Also consistent with our previous study in postmenopausal women, we observed several genes with roles in chromatin remodeling [8]. In the current study, there were four long-term upregulated genes involved in chromatin remodeling: APOBEC3G, TOX, UHRF1, and NAP1L2. These genes interact with chromatin, either by binding with histones or recruiting histone modifiers, influencing cell proliferation and differentiation among other biological processes [46–52]. In addition, APOBEC3G (apolipoprotein B mRNA-editing enzyme, catalytic polypeptide-like 3G) is involved in RNA editing [53], and deletion in APOBEC3 gene has been correlated to breast cancer risk [54]. The upregulation of these genes in the breast of P women years after delivery indicates chromatin remodeling is enabling a permanent differentiation of the breast epithelial cells.

We also evaluated whether selected genes from this study are modified in breast cancer cases. Of the genes evaluated, BHLHE22 was the most commonly modified in breast cancer (6.9% of the cases). The other genes were altered in a small percentage of breast cancer cases (Table 6). Of interest is that patients who have some alteration (amplification, deep deletion, or missense mutation) in the tested genes have a lower overall survival (Fig. 6). Although this analysis can indicate that these genes are associated with breast cancer, our interpretation is limited because the parity status of these breast cancer cases is not available.

In addition to genes involved in development and chromatin remodeling, we observed a large number of genes known to participate in immune response. The immune system has several roles in the mammary gland, being important not only for protection against pathogens, but also it is secreted into the milk and participates at the different stages of the gland development, including involution [55–58]. Of great interest, most of these immune-related genes were only upregulated in the biopsies collected within 5 years since pregnancy. This observation is consistent with our previous work in postmenopausal women, in which we did not observe enrichment of immune response in an older population [6, 7]. It is also in agreement with previous studies on molecular profiling of pregnancy performed in younger populations that reported changes in immune-response genes [59, 60].

Previous studies have showed differences in expression patterns of immune-related genes at distinct mammary developmental stages, before and/or after pregnancy in humans [59] and rodents [61, 62]. Rodent studies demonstrated that in the first days of mammary gland involution there is activation of genes related to acute-phase response and inflammation [61, 62], followed by activation of monocyte and lymphoid chemokines and immunoglobulin genes [61]. The inflammatory-like environment observed during the breast involution has been proposed as one of the mechanisms that could explain the transient increase in breast cancer risk observed after each pregnancy [57, 63]. In this study, we observed that most of the genes that underwent transient changes in expression enriched biological processes related to activation and development of lymphocytes, mainly T cells (Additional file 2). Only one GO term related to inflammation (GO:0006925: inflammatory cell apoptotic process) was enriched among the transient genes (Additional file 2). This may be due to the fact that biopsies were collected at least 1 year after FTP and/or breastfeeding; thus, we may have missed an early inflammation stage. Both human and murine postpartum mammary glands have an organized influx of immune cells; however, these cells are not observed after 1 year postpartum in human, or 12 weeks in murine [57]. We have performed immunohistochemistry reactions for dendritic cells and T cell activation markers on a subset of our samples. Using antibodies anti-LAMP3 and anti-CD123 for dendritic cells, antigen presenters, we detected few positive cells and no differences between P and NP samples (data not shown). When using anti-CD2 and anti-CD3D, markers of T cell activation, we observed an increase in cells positive for CD3D in P breast. This data suggest that there are differences in the number of activated T cells between P and NP breast (Fig. 7).

Normal pregnancy is characterized by an early expansion of regulatory T cells [64], which modulate immune tolerance during pregnancy [64]. In addition, microchimeric cells of fetal origin that persist in the maternal circulation after delivery are postulated to provide immune surveillance with the contribution of T cells, protecting against breast cancer [65–67]. Evidence shows that microchimeric cells are more frequent in healthy women than in breast cancer-affected women, and breast cancers without circulating microchimeric cells are more aggressive [65–70]. In this work, we found several upregulated genes related to activation of T cells after FTP (Additional files 2, 3, and 4). This suggests that the activation of T cells in the breast tissue of P women could trigger an early response against transformed cells, destroying them and protecting the mammary gland from neoplastic transformation. Tumor surveillance by the immune system and its impact on disease outcomes in cancer patients, and in breast cancer patients in particular, have been documented [71–75]. Finak et al. studied the stromal gene expression and clinical outcomes in breast cancer and observed that the gene set expressed predominantly in the good-outcome cluster was enriched by elements of the T helper type 1 [71]. Eight (CD2, CD8A, XCL1, CD3D, GZMA, CD247, CD48, CD52) of the genes present in this cluster [71] were also upregulated in the parous women. Poor-outcome cluster [71] showed downregulation of CXCL14, which was upregulated in the parous breast, and upregulation of CXCL1 and EDN1, which were downregulated in the parous breast. This overlap between Finak study and ours indicates that the activation of the T cell response is a beneficial mechanism against transformed cells. Winslow et al. described gene sets with better prognostics for triple-negative breast cancers [72]. These gene sets involved cytotoxic immune response, including the genes mentioned above, and HLA encoding genes [72]. HLA-DRA (major histocompatibility complex, class II, DR alpha) and HLA-DPA1 (major histocompatibility complex, class II, DP alpha 1) were constantly activated in our study. The upregulation of HLA-DPA1 has been associated with a benign behavior of certain neurological tumors and related to the immune defense-associated genes [76]. Therefore, a similar role could be attributed to the upregulation of these two genes in the cancer preventative effect of pregnancy in the premenopausal women.

Using tissue from nine women whom arrays could be conducted in epithelial tissue, stromal tissue, and whole tissue, we observed that a linear regression of gene expression in epithelial and stromal tissues predicted gene expression in the whole tissue extremely well (*R*^2^ ≥ 0.90 for all subjects). We also observed that the proportion of each tissue in the whole-tissue biopsy sample (as estimated by the linear regression coefficient) varied substantially across women (range for epithelial tissue, 0.09–0.68) (Table 7). This suggests that the P/NP gene expression differences we observed either are present in each of the two tissues (epithelial and stromal) or are present in only one of the two tissues but are of such magnitude that they are observed in whole tissue biopsies, despite the fact that some of the individual biopsies may contain only a small proportion of this tissue type.

When comparing gene expression in stroma versus epithelia, we observed a large number of genes differentially expressed among these two tissues. The epithelia, being a more differentiated tissue, enriched fewer GO terms, and these were mainly involved in epithelial development and tight junction (Additional file 7). The stroma showed enrichment of a broader range of functions, lipid homeostasis, lipid storage, metabolic process, vascularization, and migration to cite a few (Additional file 7). This extensive list of biological process is expected because the stroma is constituted of different types of cells (e.g., adipocytes, fibroblasts, endothelial cells). Among the genes found in the stroma, we did not observe enrichment of many immune-related GO terms, which is consistent with the fact we did not observe a large number of immune cells in the histopathology of these samples. Furthermore, parity status was not included in the comparison of the epithelia vs. stroma. Among the samples used in the LCM analyses (5 NP and 5 P), only one had the FTP less than 5 years before the biopsy, the group in which we found significant enrichment of immune response genes in the P/NP main study. The limited number of successful LCM microarrays did not allow us to perform analyses to understand whether parity induced more changes in expression of genes present in the stromal or epithelial cells of the breast.

This study has several strengths. A major strength is that all women were volunteers from the general population who were free of any breast abnormality. Also, all biopsies were histologically examined to confirm normality of tissue and presence of breast epithelium and stroma. Histological evaluations and gene expression experiments were performed on unidentifiable samples to prevent bias. Our study also had limitations. Because our main analysis was based on whole breast tissue microarrays, we were not able to characterize parity-associated differences in gene expression separately for epithelial and stromal tissues. While this would be of interest, it would require large biopsies in order to obtain a sufficient amount of each tissue type, which is difficult to justify in studies of healthy volunteers. The variability in composition of breast tissue among women adds to the challenge of understanding the mechanisms responsible for the preventive effect of parity and comparison of different studies. Because of the limited sample size, we could not conduct our analysis by time since last pregnancy partitioning our data in discovery/validation. Lastly, these results were generated based on a relatively homogenous population; therefore, confirmation of these results including a more ethnically heterogeneous population is needed.

## Conclusions

Altogether, parous premenopausal breast shows a specific transcriptome profile, in which genes controlling chromatin remodeling and cell differentiation are activated after FTP and stay upregulated for many years, supporting the data from postmenopausal parous women previously published [6–8].

Of great novelty is that this work shows that the genes involved in immune response were in majority related to T cell activation and these were activated soon after the FTP; however, their expression return to levels similar to those observed in the nulliparous breast five or more years after FTP. These transcripts may work in protecting the mammary gland against neoplastically transformed cells through T cells. However, because this immune surveillance appears transitory, we infer that cell differentiation, activated by the genes whose expression was permanently affected by parity, may be the main molecular mechanism responsible for the preventive effect of parity against breast cancer.

## Additional files

Additional file 1: Complete list of differentially expressed probesets (416 probes). (XLSX 101 kb)

Additional file 2: Complete list of GO terms enriched by the transient genes. (XLSX 23 kb)

Additional file 3: Complete list of GO terms enriched by the long-term changing genes. (XLSX 16 kb)

Additional file 4: Complete list of GO terms enriched by the long-term constant genes. (XLSX 20 kb)

Additional file 5: Real-time RT-PCR raw data. (XLSX 17 kb)

Additional file 6: Genes differentially expressed between epithelial and stromal tissues. (XLSX 189 kb)

Additional file 7: Complete list of GO terms enriched by the genes differentially expressed in epithelial and stromal tissues. (XLSX 61 kb)

## Abbreviations

BMI: Body mass index
CV: Coefficient of variation
FDR: False discovery rate
FTP: Full-term pregnancy
GO: Gene Ontology
IPA: Ingenuity® Pathways Analysis
LCM: Laser capture microdissection
NP: Nulliparous
NUSE: Normalized Unscaled Standard Error
P: Parous
PLM: Probe-level models
QC: Quality control
RMA: Robust Multi-Array Average
RT-PCR: Reverse transcription polymerase chain reaction
TSLP: Time since last pregnancy
WT: Whole tissue
